# Syntrophic Growth of *Geobacter sulfurreducens* Accelerates Anaerobic Denitrification

**DOI:** 10.3389/fmicb.2018.01572

**Published:** 2018-07-17

**Authors:** Yuxuan Wan, Lean Zhou, Shu Wang, Chengmei Liao, Nan Li, Weitao Liu, Xin Wang

**Affiliations:** ^1^MOE Key Laboratory of Pollution Processes and Environmental Criteria, Tianjin Key Laboratory of Environmental Remediation and Pollution Control, Nankai University, Tianjin, China; ^2^School of Environmental Science and Engineering, Tianjin University, Tianjin, China

**Keywords:** anaerobic denitrification, syntrophic growth, aggregation, *Geobacter sulfurreducens*, interspecies electron transfer

## Abstract

Nitrate is considered as a contamination since it’s over discharging to water incurs environmental problems. However, nitrate is an ideal electron sink for anaerobic pollutant degraders desiring electron acceptors due to the high redox potential. Unfortunately, not all degraders can directly reduce nitrate, and the anaerobic direct interspecies electron transfer (DIET) between degraders and denitrifiers has not been confirmed yet. Here we demonstrated that syntrophic growth of *Geobacter sulfurreducens* PCA with denitrifying microbial community at anaerobic condition eliminated the lag phase of 15 h and improved the denitrification rate by 13∼51% over a broad C/N ratio of 0.5 to 9. Quantitative PCR revealed that *G. sulfurreducens* selectively enhanced the expression of *nirS* coding for a cytochrome cd1-nitrite reductase, resulting in a fast and more complete denitrification. *Geobacter* also selectively enriched its potential denitrifying partners – *Diaphorobacter*, *Delftia*, and *Shinella* – to form spherical aggregates. More studies of the binary culture system need to be carried out to confirm the syntrophic mechanism of *Geobacter* and denitrifiers in the future. These findings extend our knowledge on understanding the anaerobic bacterial interspecies electron transfer in the denitrification process, which has broader implications in fast selection and stabilization of denitrifiers in wastewater treatment plant, and general understanding of ecology for nitrogen and metal cycling.

## Introduction

Interspecies electron transfer is critical in anaerobic digester due to the lack of electron acceptors. Syntrophic partners usually grow together to overcome energy barriers and share metabolic electrons during complex organic pollution degradation (Boone et al., 1989; Jackson and McInerney, 2002; Ishii et al., 2005). Microorganisms grown in syntrophic community transfer electrons via a few simple substrates including H_2_, formate, methanol, and acetate (Stams and Plugge, 2009). Recently, the direct interspecies electron transfer (DIET), an new electron transfer mode of microorganisms initially demonstrated in cocultures of *Geobacter* species (Rotaru et al., 2014; Lovley, 2017), is increasingly being recognized as an important way of sharing electrons when exoelectrogens are found in syntrophic community although the details and exact mechanisms still need further studies. Electrons were demonstrated to transfer directly from *Geobacter metallireducens* to *Geobacter sulfurreducens* in cocultures with ethanol as the electron donor and fumarate as the electron acceptor (Summers et al., 2010). Studies with mutant strains of *G. sulfurreducens*, as well as genome-wide transcriptomic analysis, demonstrated that H_2_ or formate is not their interspecies electron carrier. However, *Pseudomonas aeruginosa* prefer to transfer electrons to *G. sulfurreducens* via hydrogen or formate (Rotaru et al., 2012; Semenec et al., 2018). The above mentioned syntrophic partners often gather together to form aggregates to minimize the distance of adjacent cells to improve mass transfer or electron transfer.

Nitrate is a natural electron acceptor in wastewater with high redox potential of + 0.433 V to nitrite versus standard hydrogen electrode. The use of electrons from organic pollutants to reduce nitrate has a great value in terms of wastewater treatment. However, not all microorganisms are able to reduce nitrate to produce adenosine triphosphate (ATP) for their growth. Only bacteria namely denitrifiers with nitrate reductase (NAR), nitrite reductase (NIR), nitric oxide reductase (NOR), or nitrous oxide reductase (N_2_OR) are able to reduce nitrate and release molecular nitrogen (N_2_) or nitrous oxide (N_2_O) (Zumft, 1997). *G. sulfurreducens* grown on acetate was not able to reduce nitrate. It had been demonstrated that the syntrophic growth of *G. sulfurreducens* and *Wolinella succinogenes* (not use acetate) can oxidize acetate and reduce nitrate to ammonium efficiently. The electrons from acetate oxidation was transferred probably through interspecies hydrogen transfer (0.02 to 0.04 Pa) to *W. succinogenes* for nitrate reduction (Cord-Ruwish et al., 1998). However, this process is called “dissimilatory nitrate reduction to ammonium (DNRA),” which is different from the denitrification with N_2_ or N_2_O as the end product. So far, interspecies electron transfer between *Geobacter* and denitrifier was only found between *G. sulfurreducens* and an autotrophic *Thiobacillus denitrificans* when conductive nano-Fe_3_O_4_ was added (Kato et al., 2012). Unfortunately, this coculture seems to experience a DNRA process with ammonium as the end product. Nitrate cannot be reduced when Fe_3_O_4_ was removed, indicating that the denitrification between *Geobacter* and denitrifier is still not confirmed. Our hypothesis is that the denitrification through interspecies electron transfer may play a key role in mixed community when the growing condition was significantly changed, because the interspecies electron transfer, either through interspecies hydrogen transfer or DIET, is believed as an efficient route for different species to balance energy (Lovley, 2017).

Here *G. sulfurreducens* PCA was added into a denitrifying microbial community when the oxygen concentration and the carbon source were simultaneously changed. Pink aggregates were observed in cocultures, indicating the syntrophic growth. The mechanism was further explored according to the nitrate and nitrite removal, reverse transcription quantitative PCR and illumina sequencings. Denitrification at different C/N ratio was also investigated using this syntrophic community.

## Materials and Methods

### Microbial Growth and the Experimental Design

Denitrifying microbial community was acclimated from activated sludge collected from beer brewery wastewater treatment plant (Snowflake Brewery Co., Ltd., Tianjin, China) with methanol as the sole carbon source. The basal medium used for acclimation contained: Na_2_HPO_4_ 0.8 g/L, CaCl_2_ 0.1 g/L, FeSO_4_⋅7H_2_O 0.07 g/L, and MgSO_4_⋅7H_2_O 0.1 g/L. 20 mM methanol and 3∼9 mM nitrate were supplemented as the electron donor and acceptor. Activated sludge was acclimated at anoxic under shaking conditions (150 rpm) in the growth medium at 30°C. The medium was refreshed every 2 days (10% v/v) until the denitrification performance was stable (Supplementary Figure S1). The whole acclimation required 18 days. This community was then consecutively transferred for 3 batches before stored at -80°C with phosphate buffer and glycerin. In order to ensure the reproducibility of each tests, this denitrifying microbial community was reactivated from the same batch of frozen stock. The strain *G. sulfurreducens* PCA (ATCC 51573) was from laboratory frozen stocks, which was incubated in sterile anaerobic bottles (100 mL in capacity) before use.

Both the denitrifying microbial community (70 mL, OD_600_ = 0.32) and *G. sulfurreducens* PCA cells (100 mL, OD_600_ = 0.2) were collected at logarithmic growth phase by centrifugation (4000 rpm, 5 min) and washed twice to resuspend to 70 mL of anaerobic sterile medium to reach an OD_600_ of 0.6 (marked as group G-D). The medium (per liter of distilled water) contains 0.1 g KCl, 1.5 g NH_4_Cl, 0.69 g NaH_2_PO_4_⋅2H_2_O, 0.075 g CaCl_2_, 0.1g MgCl_2_⋅6H_2_O, 5 mL vitamin solution, and 12.5 mL trace minerals (Lovley and Phillips, 1988). 13 mM acetate and 3.7 mM nitrate (6 mg C/mg N to offer enough carbon for denitrification) were added as the electron donor and acceptor. It was flushed using N_2_/CO_2_ (80:20) gas for 30 min to remove oxygen, sealed with butyl rubber septa and aluminum cover, then sterilized at 121°C for 20 min. Denitrifying bacteria (marked as Group D) and *G. sulfurreducens* PCA (marked as Group G) were individually inoculated to the same anaerobic medium (OD_600_ = 0.6) as the controls. In order to confirm that the changes of denitrification performance were due to the living *G. sulfurreducens* PCA but not its intercellular or extracellular components, *G. sulfurreducens* PCA inactivated by ultraviolet radiation (10 h) or ultrasonic fragmentation (300 W, 20 min) was also added with denitrifying bacteria into anaerobic medium (**Table 1**). For the C/N ratio tests, denitrifying bacteria and denitrifying bacteria + *G. sulfurreducens* PCA were parallelly grown at C/N of 0.5, 1, 3, 6, and 9. All these experiments were carried out in a glove box (1029, Thermo Scientific, United States) in triplicate.

**Table 1:** The list of experimental groups.

Group	D	G	G-D	G-D 1	G-D 2
Denitrifiers	+	–	+	+	+
*Geobacter*	–	+	+	+ (UV sterilization)	+ (Ultrasonic fragmentation)


*“+” and “–” denote the presence and absence of *Geobacter sulfurreducens* PCA or denitrifying microbial community.*

### Nitrogen, Acetate Analysis and Aggregates Imaging

Samples were taken from each anaerobic bottle with sterile syringes at different time points, and then filtrated through 0.22 μm filter before the measurement of ammonium, nitrate, and nitrite. Nitrate and ammonium concentrations were measured by UV spectrophotometry and Nessler reagent spectrophotometry, respectively, using a UV-Vis spectrophotometer (T6, Purkinje General Instrument Co., Ltd., China) (Thomas and Cerdà, 2007). Nitrite was tested by Griess reagent spectrophotometry using multimode microplate reader (SPARK 10M, TECAN, Switzerland). Acetate was measured by high performance liquid chromatography (1525, Waters, China).

Aggregates were carefully collected from the anaerobic bottles. They were fixed with 2.5% (wt/vol) glutaraldehyde on the glass plate, and then dehydrated using a gradually increased ethanol solution from 50 to 100%. The dried samples were coated with gold, and imaged using scanning electron microscope (SEM, Shimadzu SS-550, Japan).

### Molecular Microbial Analysis

Cell density (OD_600_) was measured by multimode microplate reader (SPARK 10M, TECAN, Switzerland) based on the absorbance at 600 nm. The content of protein was measured using a BCA protein assay kit (Solarbio, Beijing).

Genomic DNA was extracted at 0, 12, 24, and 48 h from the culture of group D and G-D (6 mg C/mg N) using the Soil Genomic DNA Kit (CW2091S, ComWin Biotech Co., Ltd., China) according to the standard protocol (Feng et al., 2016). Genomic DNA of the aggregates was also extracted for analysis. The extracted DNA was sent to Majorbio (Shanghai, China) for PCR amplification. Sequencing targeted at the hypervariable V3, V4 region of 16S rDNA with the universal primers 338F and 806R, and the amplicons were subsequently determined on the Illumina MiSeq platform. R language was used for visualization of the results.

The activity of denitrifying genes (*nirK* and *nirS*) were measured using nitrite-reductase specific primers. The 876-1040 and R3cd-Cd3af primer pairs (Supplementary Table S1) were used to assess the *nirK* and *nirS* activity (Henry et al., 2004; Throback et al., 2004; Srinivasan and Butler, 2017). 16S rRNA gene was chosen as the reference for making relative quantification. The primer pair used for 16S rRNA gene quantification was 338F and 806R. The sequence of primers was listed in Supporting Information. RNA were extracted from the culture using QIAamp RNA Blood Mini Kit (52304, QIAGEN, Shanghai, China) and treated to remove any DNA contamination. The extracted RNA were then quantified with a microplate reader (NANO Quant infinite M200PRO, Tecan, Switzerland). mRNA was then converted into cDNA and stored at -20°C. Amplification of cDNA templates was carried out with Real Time PCR System (ViiA 7, Applied Biosystems, China) using SYBR Green as detection system in a reaction mixture of 16 μL containing: 0.2 μL of each primer, 8 μL of 2 × SYBR Green PCR master mix, 1 μL of the template cDNA, and RNase-free water to make up to 16 μL. All primer pairs amplifying gene fragments were then run with an initial denaturation of the DNA at 95°C for 2 min, followed by 40 cycles of 10 s at 94°C, 10 s at 60°C, and 40 s at 72°C. Triplicate wells were run for each sample for each gene target. Melting curves and negative controls were run for each qPCR run. The relative quantities of *nirK* and *nirS* transcripts were logarithmic (base 2) fold changes comparing to the value at time 0 h.

## Results and Discussion

### Denitrification Performance

**FIGURE 1:**
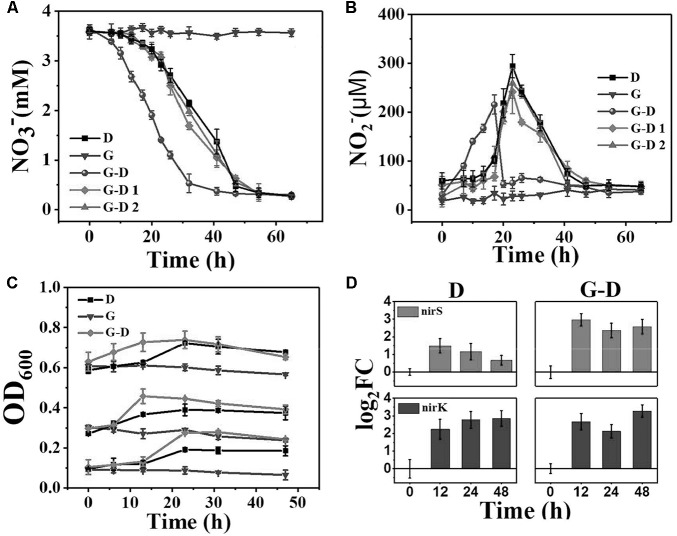
The variations of nitrate **(A)** and nitrite **(B)** concentrations in 65 h since the medium change (*t* = 0) at the C/N ratio of 6. D is denitrifying microbial community, G is the pure *G. sulfurreducens* PCA, G-D is *G. sulfurreducens* PCA added denitrifying microbial community. G-D 1 and G-D 2 are UV and ultrasonic inactivated *G. sulfurreducens* PCA added denitrifying microbial communities. **(C)** OD_600_ variations of groups D, G and G-D with initial values of 0.1, 0.3, and 0.6. Fold changes **(D)** in *nirS*, *nirK* expressions in Groups D and G-D represented as the log2 transformed value. Relative transcript ratios with 16S rRNA as reference were used to determine fold changes as compared to that of the 0 h sample. Error bars represent standard deviation from three parallel samples.

A lag period of 15 h was observed on nitrate reduction when the anoxic denitrifying microbial community was transferred to anaerobic condition with the simultaneous carbon source changing from methanol to acetate (Group D, **Figure 1A**). Nitrite, the intermediate product during denitrification, was not detected to increase at this period. The 59 ± 16 μM of nitrite at the beginning was believed to be the background value desorbed from the biomass (**Figure 1B**). After that, a linear decrease of nitrate was observed, with a nitrate removal rate of 30 g N/m^3^⋅d. Nitrite slightly accumulated at the same time, which reached the maximum of 294 ± 24 μM at 23 h.

Very interestingly, when *G. sulfurreducens* PCA was mixed with the denitrifying microbial community (Group G-D), nitrate reduction started immediately without any lag period. An adaption period is usually needed for the switch from anoxic to anaerobic growth and also from methanol as electron donor, as showed in **Figure 1A**. *Geobacter* adapted very well with the “new” niche, however, lacking of electron donor. It is hypothesized that *Geobacter* and several denitrifiers fast united as a strategy to survive through interspecies electron transfer, so that the lag phase seems to be eliminated. The denitrification rate was accelerated by 33% to 40 g N/m^3^⋅d, and the peak nitrite concentration appeared 6 h earlier with a 27% lower value (215 ± 20 μM, **Figure 1B**). The nitrite concentration peak gradually decreased to the background in 24 h in Group D, but it was suddenly dropped down in Group G-D after the peak value was achieved at 17 h, indicating that the denitrification of Group G-D was more complete than Group D. Both Groups D and G-D reached the maximum nitrate removal efficiency of 92%, however, with different times. The addition of *G. sulfurreducens* PCA shortened the time needed to reach 90% nitrate removal by 22% from 54 to 42 h. We did not find any N_2_O in the headspace, probably because N_2_O was usually produced at anoxic but not anaerobic condition (Ding et al., 2016; Yan et al., 2016). The concentration of ammonium was almost constant Group D and G, but slightly increased in Group G-D (Supplementary Figure S3), and acetate consumption matched the nitrate reduction (Supplementary Figure S4). Around 38% of nitrate was reduced to ammonium, which confirmed that the denitrification but not DNRA process dominated in this system. This is very different from previous *G. sulfurreducens* PCA involved DNRA reports (Cord-Ruwish et al., 1998; Kato et al., 2012).

The pure culture of *G. sulfurreducens* PCA (Group G) was not able to reduce nitrate to nitrite (**Figures 1A,B**), showing that all the changes between Groups D and G-D were attributed to the interaction between *G. sulfurreducens* PCA and other microorganisms. In order to confirm whether the living *Geobacter* or some components from *Geobacter* (such as redox cytochromes) mainly resulted in these changes, we mixed *G. sulfurreducens* PCA treated with ultraviolet radiation (Group G-D1) or ultrasonic fragmentation (Group G-D2) and found that both groups cannot accelerate denitrification (**Figure 1A**), demonstrated that only the enzymes, proteins or even fragments from *G. sulfurreducens* cannot accelerate the nitrate removal (**Figure 1**).

The activities of denitrifying genes were usually evaluated by quantifying the amount of NIR genes (*nirK* and *nirS*) using real-time quantitative PCR because the reduction of nitrite is usually the rate-limiting process (Throback et al., 2004). The *nirK* and *nirS* encode two types of structurally different but functionally similar NIRs [a copper nitrite reductase encoded by the *nirK* and a cytochrome cd1-NIR encoded by the *nirS* (Zumft, 1997)]. Significant increases of *nirK* and *nirS* were observed over 12 h in both Groups of G-D and D (**Figure 1D**), corresponding to the fast removal of nitrate and nitrite in the initial 48 h. The log_2_FC values of *nirK* transcripts was constant of 2.70 ± 0.57, showing an insignificant change between two groups. However, the log_2_FC values of *nirS* transcripts gradually decreased from 1.49 ± 0.40 to 0.67 ± 0.28 from 12 h to 48 h in Group D, 2∼4 times lower than that of *nirK*. The addition of *G. sulfurreducens* PCA selectively enhanced the expression of *nirS* to a comparable level of *nirK*, resulting in a fast and more complete denitrification by avoiding the accumulation of nitrite. This was in accordance with the lower peak of nitrite observed in Group G-D. It should be noted that the genome of *G. sulfurreducens* PCA does not contain *nirK* or *nirS* (Methe et al., 2003), so the enhancement should be due to the change of denitrifying microbial community. The OD_600_ presented a slight decrease of biomass in Group G, comparing to an obvious increase in the Group D and G-D, showing the growth of bacteria in both groups. Especially, the Group G-D exhibited a faster increase of OD_600_ in the initial 24 h than that of Group D, corresponding to the faster acetate and nitrate consumption (Supplementary Figure S4), indicating that the addition of *Geobacter* accelerated the bacterial growth. In order to have more information of this process, it is necessary to analyze the succession of the microbial community at different time profile.

### Reshaping of Denitrifying Microbial Community

**FIGURE 2:**
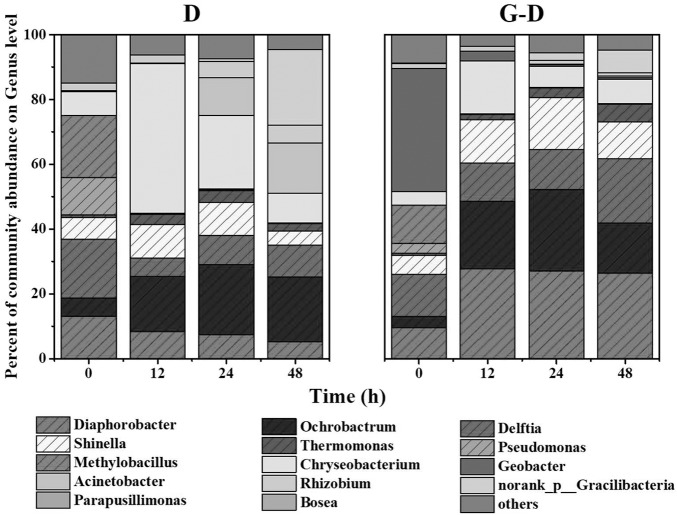
Relative abundance of microbial community at genus level in Groups D (denitrifying microbial community) and G-D (*G. sulfurreducens* added denitrifying microbial community) at different time profiles. Shaded areas represent the putative denitrifiers. Genera with relative abundance lower than 5% was classified into group “others.” All samples were collected from planktonic cells.

The denitrifying microbial community was initially acclimated at anoxic from activated sludge using methanol as the sole carbon source. According to the records in literatures (Khan and Hiraishi, 2002; Shigematsu et al., 2003; Chistoserdova et al., 2007; Bai et al., 2009), the putative denitrifying bacteria were mainly from genera of *Diaphorobacter*, *Ochrobactrum*, *Delftia*, *Shinella*, *Thermomonas*, *Pseudomonas*, and *Methylobacillus* (**Figure 2**). When it was transferred to anaerobic condition with acetate as the sole carbon source, *Pseudomonas* and *Methylobacillus* were replaced by other bacteria, probably because *Pseudomonas* was aerobic bacteria and *Methylobacillus* was putative denitrifier specifically living on methanol (Chistoserdova et al., 2007). Some of the genera were selectively enriched after *G. sulfurreducens* PCA addition. Taking samples at 12 h as an example, *Diaphorobacter* predominantly presented (26%) in Group G-D, while that genus only accounted for 5.2% in Group D. Besides, *Delftia* and *Shinella* had 20 and 11% in Group G-D, but their abundances in Group D were relatively small (10 and 4.4%). There was almost no difference between two conditions in the relative abundance of some putative denitrifiers such as *Ochrobactrum* and *Thermomonas*.

On the time scale, *Chryseobacterium* (a genus of Gram-negative bacteria) in Group D became predominant after 12 h from 7.2% (0 h) to 46%, while the growth of *Diaphorobacter* and *Delftia* were inhibited. Two minor genera of *Acinetobacter* and *Gracilibacteria* were amplified from 0.1% (12 h) to 12% (24 h) and 0.8% (24 h) to 23% (48 h). The microbial community composition was relatively stable after *G. sulfurreducens* PCA addition (G-D in **Figure 2**) in 48 h. Different from the Group D, *Chryseobacterium* were consecutively inhibited in terms of abundance from 16.3% at 12 h to 6.5% at 48 h.

In general, *G. sulfurreducens* PCA enhanced the total putative denitrifiers from 47 ± 5% to 80 ± 4%. The existence of *G. sulfurreducens* PCA was beneficial to the growth of putative denitrifiers such as *Diaphorobacter*, *Delftia*, and *Shinella* in the mixed denitrifying microbial community, even though planktonic *Geobacter* gradually decreased from 38% (0 h) to 0.4% (24 h) in 24 h. It is clear that the syntrophic growth of *G. sulfurreducens* PCA accelerated the reshaping of microbial community, constructed a stable and efficient (**Figure 1**) denitrifying community in a couple of its doubling time (12 h, the doubling time for *Geobacter* is 6 h). How the *Geobacter* interact with other microorganisms? Where are they? We observed pink aggregates in Group G-D.

### Morphology and Microbial Community of Aggregates

**FIGURE 3:**
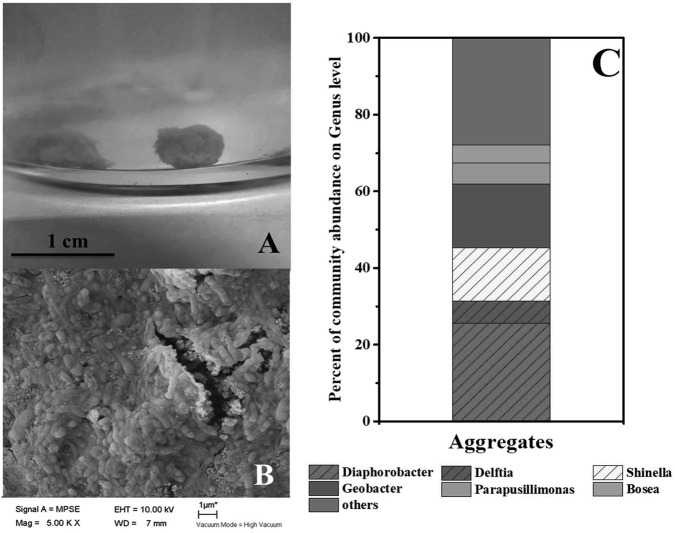
Photo **(A)** and SEM image **(B)** of aggregates in *G. sulfurreducens* PCA added denitrifying microbial community. Relative abundance of microbial community at genus level in the pink aggregates **(C)**.

As mentioned in context, syntrophic partners often gather together to form aggregates. The coculture of *G. sulfurreducens* and denitrifying microbial community formed large pink aggregates (diameter > 8 mm, **Figure 3A** and Supplementary Figure S2) in 24 h, which was different from the aggregates formed in *G. sulfurreducens/G. metallireducens* cocultures. Rod-shape bacterial cells huddled tightly to form a spherical surface, ensuring an effective electron transfer or mass transfer between adjacent cells (**Figure 3B**). It was showed that the biomass of the aggregates accounted for 45% of the total biomass (Supplementary Figure S5), showing that bacteria in Group G-D equally distributed between the aggregation and planktonic cells. Sequencing of these aggregates revealed that the *Diaphorobacter* (26%), *Geobacter* (17%) and *Shinella* (14%) were predominant genera, preliminarily demonstrated the syntrophic growth of *Geobacter* with *Diaphorobacter* and *Shinella* (**Figure 3C**). Comparing to the *Geobacter* abundance of 0.4% in suspension, *Geobacter* mainly accumulated in these aggregates. It could not be ruled out that *Delftia* was syntrophic partner even though the proportion of *Delftia* in the community was relatively small (6%).

The formation of aggregates and the significant acceleration of nitrate removal in cocultures suggested a mechanism of syntrophic interaction where *G. sulfurreducens* and denitrifiers combine their metabolism to a fast and energy efficient route to utilize acetate and reduce nitrate. It was reported that *G. sulfurreducens* produce H_2_ when the electron acceptor is limited (Cord-Ruwish et al., 1998). Therefore, the interspecies electron transfer between *Geobacter* and denitrifiers in this study probably uses H_2_ as the electron carrier. Unfortunately, the H_2_ concentration in the headspace was lower than the detecting limit in all samples, probably because the interspecies hydrogen transfer only allows a very low (0.02–0.04 Pa) H_2_ partial pressure (Cord-Ruwish et al., 1998). DIET might be the other mechanism, which has to be confirmed in binary cultures in the future.

### Denitrification at Different C/N Ratios

**FIGURE 4:**
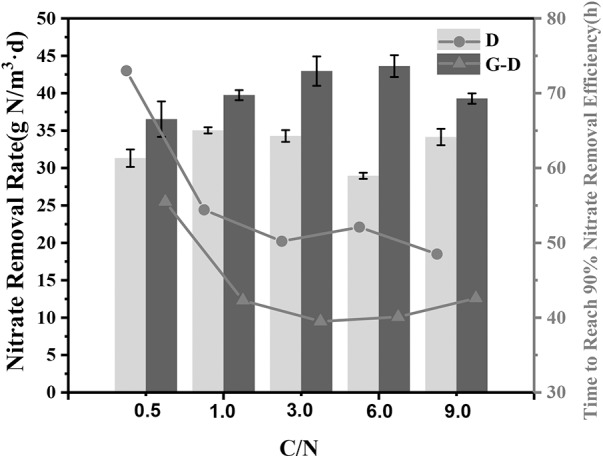
Nitrate removal rate under different C/N ratios (0.5, 1, 3, 6, and 9) in Group D (denitrifying microbial community) and G-D (*G. sulfurreducens* added denitrifying microbial community). The rate was calculated by fitting a straight line and taking the derivative for the variations of nitrate removal efficiency. The lines represent the time required to reach 90% nitrate removal efficiency.

In order to test the denitrification performance in this syntrophic system, the C/N ratio in the medium was changed to 0.5, 1, 3, 6, and 9 individually. As expected, the nitrate removal rates in all *Geobacter* added systems were 13∼51% greater than those in group D even at a very low C/N ratio of 0.5 (**Figure 4**). All G-D groups except for the C/N ratio of 0.5 spent 41 ± 1.5 h to reach 90% removal efficiency, but the denitrifying microbial community without *Geobacter* required 52 ± 3 h. At the C/N ratio of 0.5, the denitrifying microbial community needed a longer time of 73 h to reach the same nitrate removal due to the lack of carbon source, while *Geobacter* shortened the time by 25% to 56 h. The syntrophic growth of *G. sulfurreducens* PCA was further demonstrated to accelerate denitrification rate at a broad C/N ratio range from 0.5 to 9.

### Implications

Here we demonstrated that the syntrophic growth of *G. sulfurreducens* with denitrifying microbial community significantly accelerated the anaerobic denitrification process over a C/N ratio range of 0.5 to 9. *Geobacter* selectively enriched its potential partners (such as *Diaphorobacter*, *Delftia*, and *Shinella*) in mixed culture to form aggregates, and the expression of *nirS* was enhanced as a result. Our findings showed the evidence of interspecies electron transfer between exoelectrogens and denitrifiers to produce N_2_, although the exact biological process and scientific mechanism should be further explored. This is very important to understand the nitrogen and metal cycling driven by microorganisms.

Our findings can be further applied to stabilize denitrifying microbial community when the operational conditions changes in wastewaters. For example, AAO, the most widely used technology for denitrification, requires a recirculation of aerobic activated sludge back to anaerobic tank. The denitrification usually has a lag phase due to the change of oxygen and substrate. If a bioelectrochemical system is added to the anaerobic tank in the future, exoelectrogens can accelerate the denitrification process to shorten the hydraulic retention time (HRT) and narrow the volume of the tank, reducing the total cost and energy inputs for denitrification.

## Author Contributions

XW designed this experiment and revised the manuscript. YW did most of the test and wrote this manuscript. LZ and SW helped to grow *G. sulfurreducens* PCA. CL helped for RNA extraction and sequencing analysis. NL and WL helped to improve the quality of this manuscript.

## Conflict of Interest Statement

The authors declare that the research was conducted in the absence of any commercial or financial relationships that could be construed as a potential conflict of interest.
