# Supplementary material for: Syntrophic Growth of Geobacter sulfurreducens Accelerates Anaerobic Denitrification
Source: Front Microbiol. 2018 Jul 17;9:1572. doi: 10.3389/fmicb.2018.01572 (PMC6056638; doi:10.3389/fmicb.2018.01572)
Supplement: Supplementary file 1 [file Image_1.PDF]

-Supporting Information-

Syntrophic Growth of Geobacter *sulfurreducens* Accelerates Anaerobic Denitrification

Yuxuan Wan[1], Lean Zhou[1], Shu Wang[2], Chengmei Liao[1], Nan Li[2], Weitao Liu[1] and Xin Wang[1]*

[1] MOE Key Laboratory of Pollution Processes and Environmental Criteria / Tianjin Key Laboratory of Environmental Remediation and Pollution Control, Nankai University, No. 38 Tongyan Road, Jinnan District, Tianjin 300350, China
[2] School of Environmental Science and Engineering, Tianjin University, No. 92 Weijin Road, Nankai District, Tianjin 300072, China

*Corresponding author: Phone: (86)18722292585; fax: (86)22-23501117; E-mail: xinwang1@nankai.edu.cn

Table S1 The primer sequences pair used for 16S rRNA gene quantification

Primer	Sequence
nirK 876	5'-ATYGGCGGVAYGGCGA-3'
nirK 1040	5'-GCCTCGATCAGRTTRTGGTT-3'
nirS R3cd	5'-GASTTCGGRTGSGTCTTSAYGAA-3'
nirS Cd3af	5'-GTSAACGTSAAGGARACSGG-3'
338F	5'-ACTCCTACGGGAGGCAGCAG-3'
806R	5'-GGACTACHVGGGTWTCTAAT-3'

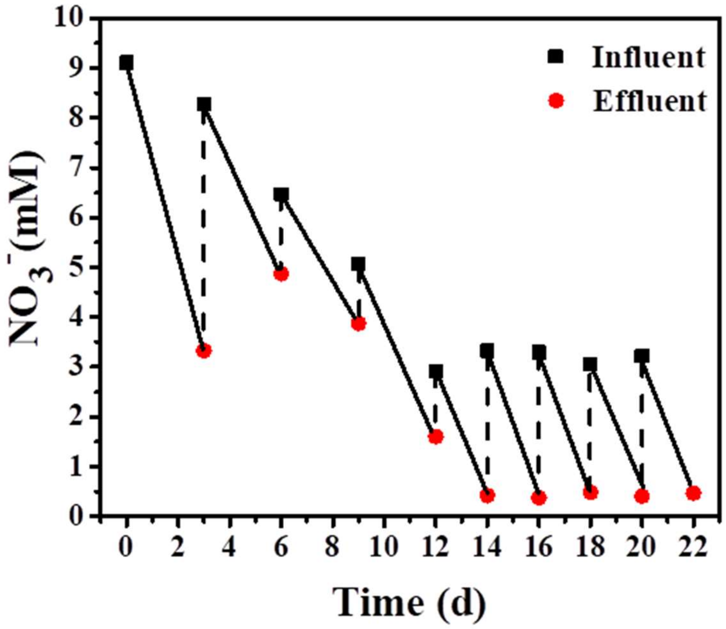

Figure S1 Variations of nitrate during acclimation. The medium was refreshed at the dotted line.

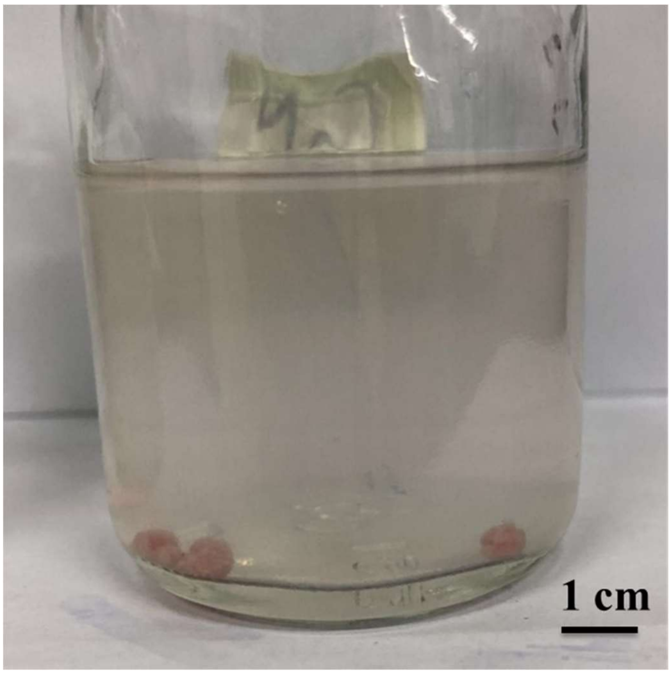

Figure S2 Spherical aggregates was formed in *G. sulfurreducens* PCA added denitrifying microbial communities**.**

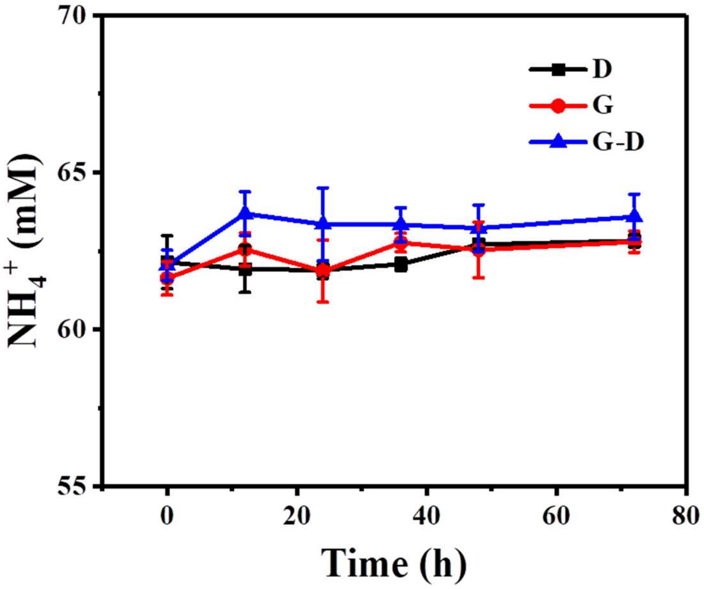

Figure S3 The variations of ammonium concentrations after the medium change (t = 0) at the C/N ratio of 6. The error bars represent the standard deviation of replicated experiments (n=3).

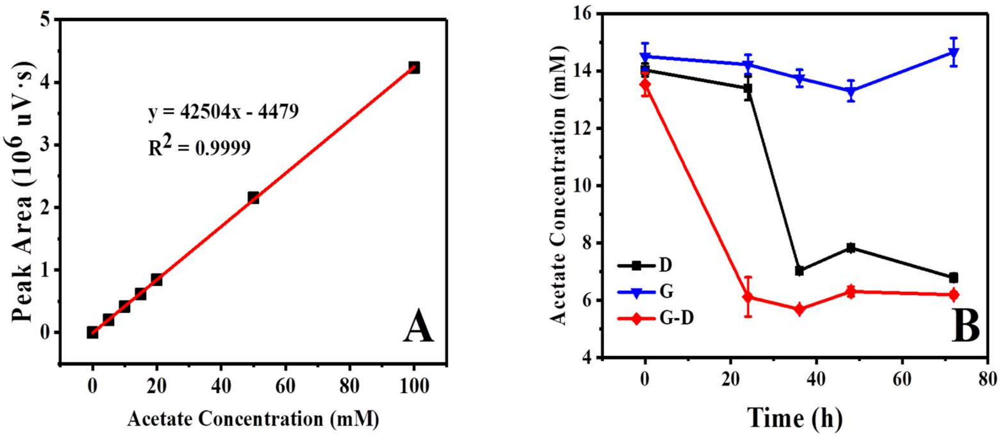

Figure S4 (A) The standard curve of acetate concentrations by high performance liquid chromatography. (B) The variations of acetate concentrations after the medium change (t = 0) at the C/N ratio of 6. The error bars represent the standard deviation of replicated experiments (n=3).

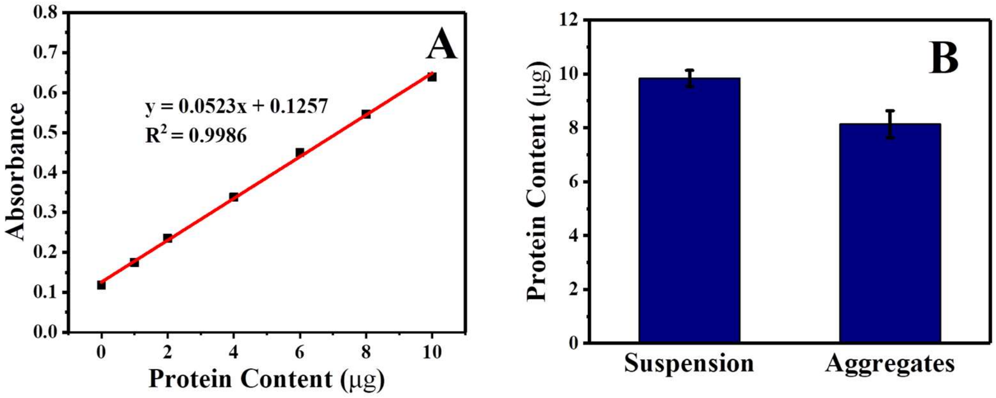

Figure S5 (A) The standard curve of protein content based on the absorbance at 562nm. (B) The protein content of suspension and aggregates in group G-D. The error bars represent the standard deviation of replicated experiments (n=3).
